# Upregulation of miR-31* Is Negatively Associated with Recurrent/Newly Formed Oral Leukoplakia

**DOI:** 10.1371/journal.pone.0038648

**Published:** 2012-06-18

**Authors:** Wen Xiao, Zhe-Xuan Bao, Chen-Yang Zhang, Xiao-Yun Zhang, Lin-Jun Shi, Zeng-Tong Zhou, Wei-Wen Jiang

**Affiliations:** Department of Oral Mucosal Diseases, Shanghai Ninth People’s Hospital, Shanghai Jiao Tong University School of Medicine, Shanghai Key Laboratory of Stomatology, Shanghai, China; The Chinese University of Hong Kong, Hong Kong

## Abstract

**Background:**

Oral leukoplakia (OLK) is a potentially malignant disorder of the oral cavity. However, the underlying mechanism of OLK is still unclear. In this study, we explore possible miRNAs involved in OLK.

**Methodology/Principal Findings:**

Using miRNA microarrays, we profiled miRNA expression in OLK and malignantly transformed OLK (mtOLK) tissue samples. The upregulation of miR-31*, miR-142-5p, miR-33a, miR-1259, miR-146b-5p, miR-886-3p, miR-886-5p, miR-519d, and miR-301a along with the downregulation of miR-572, miR-611, miR-602, miR-675, miR-585, miR-623, miR-637, and miR-1184 in mtOLK were new observations. Fluorescence *in situ* hybridization (FISH) analyses confirmed that miR-31* is highly expressed in mtOLK. There was a significant difference between the FISH score (p<0.05) in patients with or without recurrent/newly formed OLK. Functional analyses demonstrated that a miR-31* inhibitor decreased apoptosis in the Leuk-1, which is an immortalized oral epithelial cell line spontaneously derived from an oral leukoplakia lesion. miR-31* regulated apoptosis, cell proliferation, migration, and invasion in the HOIEC, which is a HPV E6/E7-immortalized oral epithelial cell line. Furthermore, miR-31* modulated the biological functions of apoptosis, cell proliferation, cell cycle, migration, and invasion in the oral squamous cell carcinoma cell line, Cal-27. Using bioinformatic analyses and dual luciferase reporter assays, we determined that the 3′ untranslated region of fibroblast growth factor 3 (FGF3) is the target of miR-31*. Expression of FGF3 was downregulated or upregulated in the presence of a miR-31* mimic or inhibitor, respectively.

**Conclusions/Significance:**

Upregulation of miR-31* is negatively associated with recurrent/newly formed OLK. MiR-31* may exert similar but distinguishable effects on biological function in oral cells with different malignant potential. FGF3 is the target of miR-31*. miR-31* may play an important role during OLK progression through regulating FGF3. MiRNA* strands may also have prominent roles in oral carcinogenesis.

## Introduction

The development of oral squamous cell carcinoma (OSCC) is considered a multistep process starting with hyperplasia, progressing to dysplasia, and finally to neoplasm. During these steps, multiple genetic alterations may occur, including chromosomal aberrations as well as DNA mutations, amplification, or deletions [1]. Therefore, oral leukoplakia (OLK) is recognized as an excellent research model for oral carcinogenesis. OLK is a potentially malignant disorder of the oral cavity, and presents as “a predominantly white lesion of the oral mucosa that cannot be characterized as any other definable disease” [2]. There are two main clinical types of OLK, homogeneous and non-homogeneous, that may affect any site of the oral cavity as a single, multiple, or diffused lesion. Some OLKs resist treatment and exhibit local recurrence (up to 30%) [3] or malignant transformation [4]. In spite of tremendous progress in the field of molecular biology, the underlying mechanism of OLK is still poorly understood.

MicroRNAs (miRNAs), a novel class of small non-coding RNAs composed of 19–25 nucleotides, can post-transcriptionally regulate gene expression by degrading mRNA or repressing translation [5]. More than one-quarter of all known human miRNAs are significantly dysregulated in at least one cancer type, suggesting that miRNAs may represent one of the largest classes of gene regulators in cancer-related processes [6]. Several putative oncogenic miRNAs have been identified in head and neck squamous cell carcinoma, including miR-21, miR-184, and miR-31 [7], [8], [9].

miRNA precursors, pre-miRNAs, are comprised of two strands: the leading strand used for the production of mature miRNA and the passenger strand (termed miRNA*) that is believed to be degraded [10]. However, recent studies have reported that many miRNA* species are in fact relatively abundant and physically associate with effector complexes [11], [12], [13]. However, there is limited information about miRNAs* in relation to cancer.

In this study, we employed miRNA microarrays to identify global changes of miRNA expression in OLK and malignantly transformed OLK (mtOLK) tissue samples. We found 25 upregulated miRNAs and nine downregulated miRNAs with greater than 2-fold changes in mtOLK. Fluorescence *in situ* hybridization (FISH) was then used for further verification of one of the significantly altered miRNAs, miR-31* and confirmed that miR-31* was overexpressed in mtOLK. Functional analyses indicate that miR-31* may exert similar but distinguishable effects on biological function in oral cells with different malignant potential. Furthermore, using bioinformatic analyses and dual luciferase reporter assays, we demonstrated that the 3′-untranslated region (UTR) of fibroblast growth factor 3 (FGF3) is the target of miR-31*. We also investigated changes in FGF3 expression in the presence of miR-31* mimics/inhibitors. Our findings suggest miR-31* dysregulation is involved in the OLK progression through regulating FGF3. miRNA* strands may also have prominent roles in oral carcinogenesis.

## Results

### miRNA Expression Patterns Vary Significantly between OLK and mtOLK

**Figure 1:**
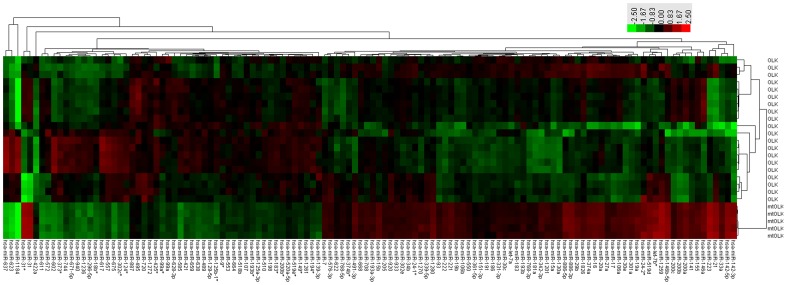
Unsupervised hierarchical cluster analysis of miRNA expression in OLK and mtOLK. The marked separation of OLK and mtOLK based on the miRNAs profiling is exhibited. Scale is shown in the upper right. Red represents a high expression and green represents a low expression. OLK, oral leukoplakia; mtOLK, malignant transformed oral leukoplakia.

As compared with OLK tissue samples, there were 72 upregulated and 50 downregulated miRNAs found in mtOLK (p<0.01). Unsupervised hierarchical clustering analyses of the 122 miRNAs revealed a marked separation of mtOLK and OLK, suggesting this differential expression profile could be used as a possible phenotypic discriminator between OLK and mtOLK (Figure 1). Of the 122 altered miRNAs, 25 upregulated miRNAs and nine downregulated miRNAs exhibited a greater than 2-fold change in expression. As previously reported in other cancers [14], [15], [16], [17],[18],[19],[20],[21], we observed upregulation of miR-142-3p, miR-223, miR-31, miR-21, let-7b*, miR-19a, miR-200a, miR-200b, miR-30e, miR-146a, miR-141, miR-222, miR-374a, miR-221, miR-24-2*, and miR-16 as well as downregulation of miR-373*. However, the upregulation of miR-31*, miR-142-5p, miR-33a, miR-1259, miR-146b-5p, miR-886-3p, miR-886-5p, miR-519d, and miR-301a along with the downregulation of miR-572, miR-611, miR-602, miR-675, miR-585, miR-623, miR-637, and miR-1184 were new observations. Importantly, we noticed that miR-31* and miR-31, which are derived from the same precursor, are both upregulated in mtOLK.

### Verification of miRNA Expression using FISH Analyses

Next, we performed FISH to verify and localize miR-31* expression in 10 OLK and five mtOLK tissue samples. A positive fluorescent miR-31* signal was found in 1/10 (10%) of OLK patients and 4/5 (80%; p>0.05) of mtOLK patients. Interestingly, one of the OLK tissue samples that was positive for miR-31* was identified as severe dysplasia. Furthermore, the FISH score was higher in mtOLK (mean = 1.2) than in OLK (mean = 0.2, p<0.05; Table 1). In addition, no statistically significant differences were found between a positive fluorescent miR-31* signal and gender, age, epithelial dysplasia, or bad habits such as smoking and excessive drinking and eating spicy/hot food and betel nut chewing.

**Figure 2:**
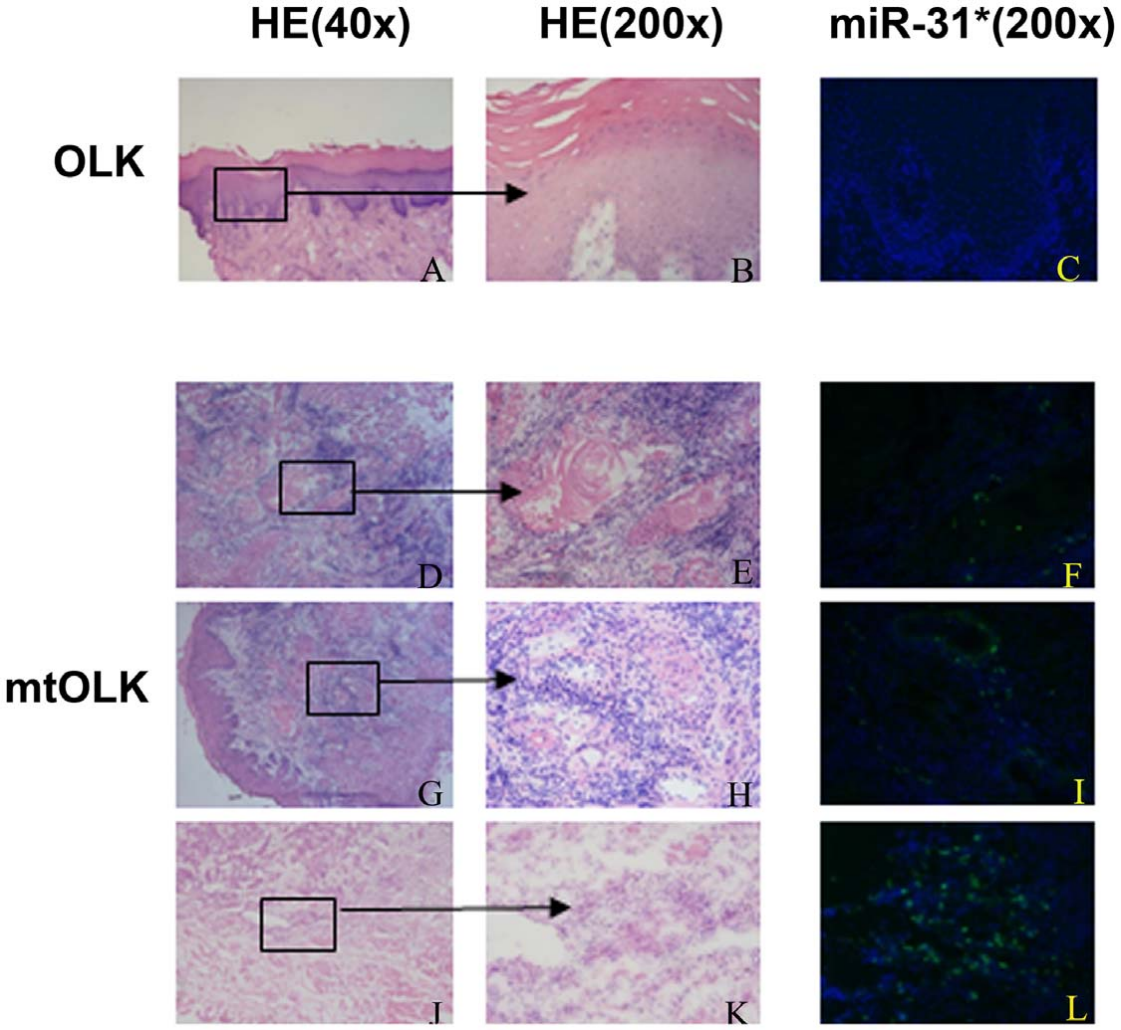
FISH detection of miR-31* expression in OLK and mtOLK. (**A**) ∼ (**C**), no positive signal is detected in OLK. (**D**) ∼ (**L**), the representative positive signals are showed in mtOLK. (**D**) ∼ (**F**), tumor nest; (**G**) ∼ (**I**), vascular area; (**J**) ∼ (**L**), inflammation. FISH signals are visualized in green, while blue depicts nuclear DAPI stain. OLK, oral leukoplakia; mtOLK, malignant transformed oral leukoplakia.

We also noticed that the unique OLK tissue sample expressing miR-31* was negative for recurrent or newly formed OLK during the follow-up. Meanwhile, the one mtOLK that had a FISH score of 0 for miR-31* was found to have a newly formed OLK lesion during the follow-up (Table 1). Therefore, we performed a univariate regression model to analyze the relationship between recurrent or newly formed OLK and such parameters as gender, age, epithelial dysplasia, bad habits, malignant transformation, and FISH scores. Only the FISH score (p<0.05) presented a significant difference. In addition, miR-31* was mainly detected in the inflammatory region, vascular area, and cancer nest of mtOLK (Figure 2).

**Table 1:** Clinical outcomes in OLK and mtOLK.

ID	Lesion	Epithelialdysplasia	FISHscore	Recurrent	Newlyformed	Follow-up (months)
1	OLK	ND	0	NA	NA	27
2	OLK	MiD	0	(+)	(−)	47
3	OLK	MiD	0	NA	NA	29
4	OLK	MiD	0	(+)	(−)	20
5	OLK	MoD	0	(+)	(−)	37
6	OLK	MoD	0.	(−)	(−)	30
7	OLK	SD	0	(+)	(−)	42
8	OLK	SD	0	NA	NA	32
9	OLK	SD	0	(−)	(−)	29
10	OLK	SD	2	(−)	(−)	30
11	mtOLK	SCC	0	(−)	(+)	36
12	mtOLK	SCC	2	(−)	(−)	28
13	mtOLK	SCC	1	(−)	(−)	33
14	mtOLK	SCC	1	NA	NA	21
15	mtOLK	SCC	2	(−)	(−)	21

OLK, oral leukoplakia; mtOLK, malignant transformed oral leukoplakia; ND, no dysplasia; MiD, mild dysplasia; MoD, moderate dysplasia; SD, severe dysplasia; SCC, squamous cell carcinoma; NA, not available.

### The Effect of miR-31* on the Biological Function of Oral Cell Lines with Different Malignant Potential

**Figure 3:**
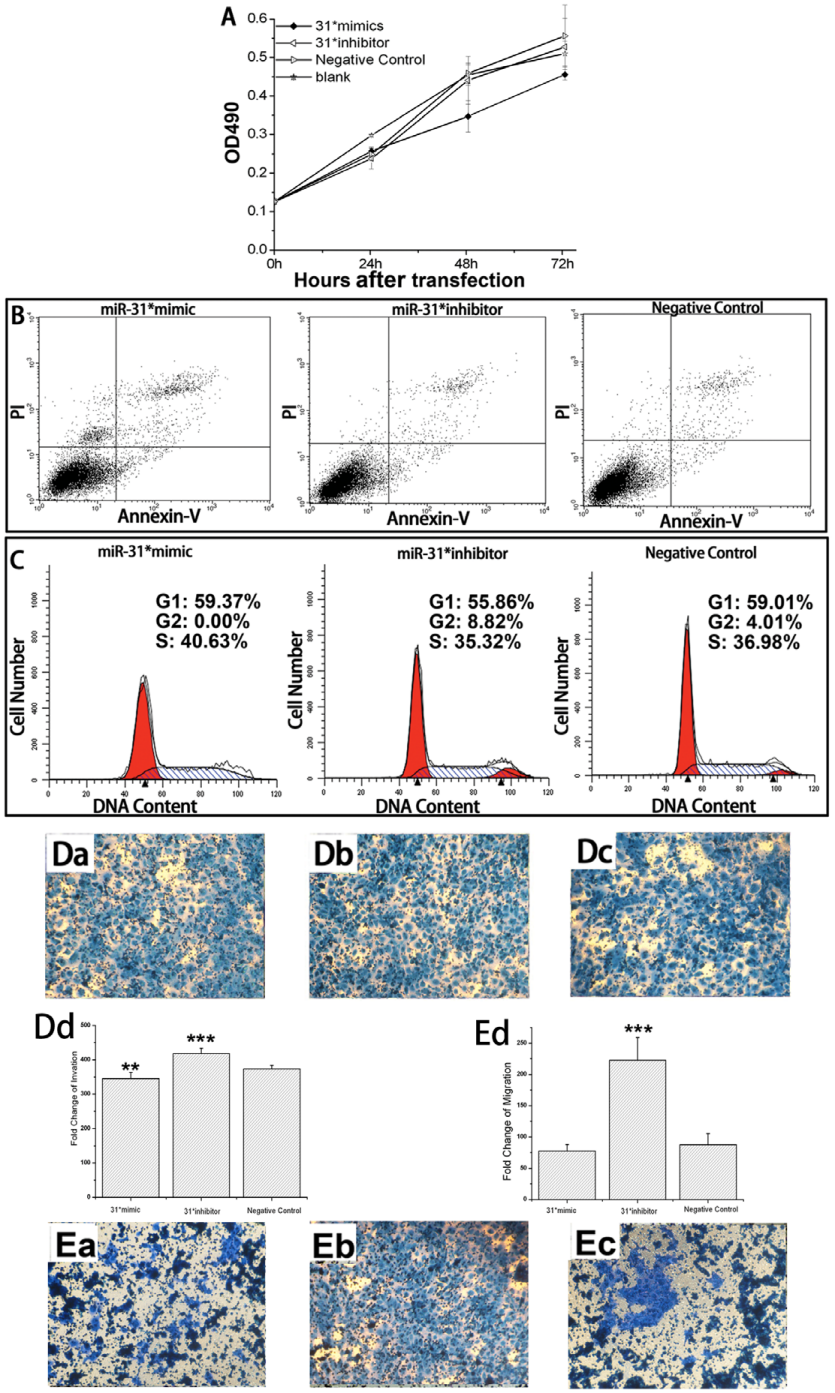
Representative results of miR-31* effects on biological functions. (**A**) the MTT assay in HIOEC; (**B**) the Annexin V assay in Leuk-1; (**C**) flow cytometry analysis for cell cycle in Cal-27; (**D**) representative field of view of HIOEC invasion inserts at a 100× magnification, (Da) miR-31* mimic, (Db) miR-31* inhibitor, (Dc) negative control, (Dd) Quantification of relative numbers of invading cells representing average counts from 6 fields-of-view per insert per sample ±SD, **p<0.05, ***p<0.001; (**E**) representative field of view of HIOEC migration inserts at a 100× magnification, (Ea) miR-31* mimic, (Eb) miR-31* inhibitor, (Ec) negative control, (Ed) quantification of relative numbers of invading cells representing average counts from 6 fields-of-view per insert per sample ±SD, **p<0.05, ***p<0.001.

Leuk-1 is an oral keratinocyte cell line with an immortalized but non-tumorigenic phenotype. This cell line is spontaneously derived from an oral leukoplakia lesion adjacent to OSCC [22]. After transfection with the miR-31* mimic, the number of apoptotic cells increased by 30.5% (p<0.05). However, in the presence of the miR-31* inhibitor, the percentage of apoptotic Leuk-1 cells decreased by 11.2% (p>0.05). The migration and invasion abilities of Leuk-1 cells transfected with the miR-31* mimic decreased by 24.1% (p<0.05) and 33.8% (p<0.05), while in the presence of the miR-31* inhibitor the migration and invasion abilities increased by 80.45% (p<0.0001) and 3.38% (p>0.05), respectively. miR-31* had only limited effects on cell proliferation as well as cell cycle (Figure 3 and Figure S1).

HIOEC is an immortalized oral keratinocyte cell line induced with HPV16 E6/E7 [23]. Subsequent treatment of HIOEC with benzol[a]pyerene for 6 months resulted in the development of a cancerous cell line [24]. The percentage of apoptotic cells was slightly decreased (4.4%) in HIOEC cells in the presence of the miR-31* inhibitor. Cell proliferation in the presence of the miR-31* mimic was inhibited by 11.09% as compared with the negative control. The migration and invasion abilities of HIOEC cells transfected with the miR-31* inhibitor significantly increased by 154.2% (p<0.0001) and 12% (p<0.05). No significant effect of miR-31* on cell cycle was found in HIOEC cells (Figure 3 and Figure S2).

Proliferation of the OSCC cell line Cal-27 was inhibited 9.66% (p>0.05) and the number of apoptotic cells increased 5.5% (p<0.05) in the presence of the miR-31* mimic. The percentage of Cal-27 cells in the S/G2 phase of the cell cycle increased by 7.6% (p<0.05) upon transfection with the miR-31* inhibitor. The migration and invasion abilities of Cal-27 cells transfected with the miR-31* inhibitor significantly increased by 35% (p<0.05) and 21% (p<0.05), respectively (Figure 3 and Figure S3).

### FGF3 is a Target of miR-31*

**Figure 4:**
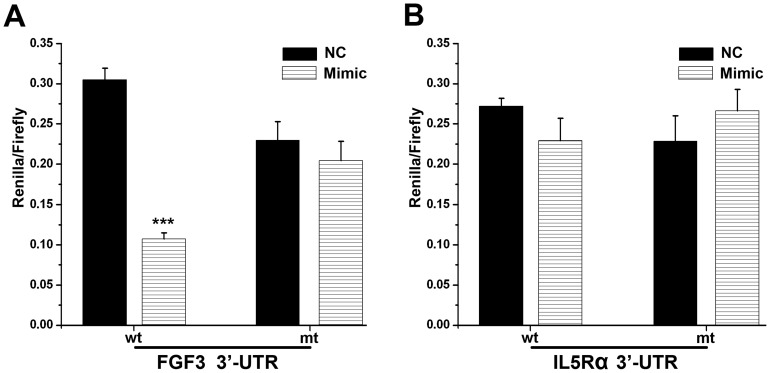
Dual luciferase reporter assay for miR-31* and targeted genes. (**A**) Psicheck-FGF3-wt/mt and miR-31*; (**B**) Psicheck-IL5Rα-wt/mt and miR-31*. NC  =  negative control; mimic  =  miR-31* mimic; wt  =  wild type; mt  =  mutant type.

**Figure 5:**
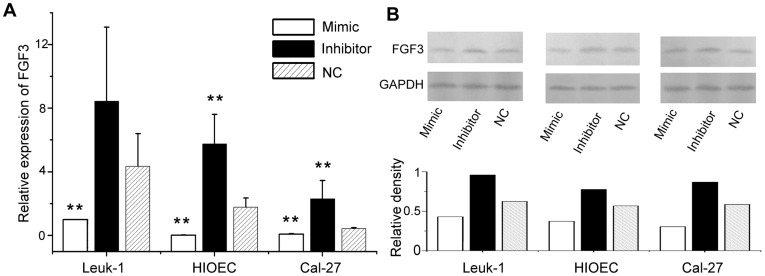
miR-31* mimics/inhibitors regulating mRNA and protein expression of FGF3 in oral cells. (**A**) QRT-PCR analysis of FGF3 expression in Leuk-1, HIOEC and Cal-27 cell lines, **p<0.05; (**B**) Western blot analysis of of FGF3 expression in Leuk-1, HIOEC and Cal-27 cell lines transfected with miR-31* mimic, miR-31* inhibitor and negative control. The representative blot is shown (upper for FGF3). The histogram shows the average volume density normalized by the loading control, GAPDH (lower).

Four algorithms (PicTar, TargetScan, miRanda, and MicroCosm Targets) were used to predict the putative targets of miR-31*. Only MicroCosm Targets showed the candidate targets of miR-31*, which included 655 genes (Table S1). Of these genes, FGF3 and the interleukin 5 receptor α (IL5Rα) were selected for further validation. We cloned the 3′ UTR fragments of human FGF3 or IL5Rα containing a wild type or mutant miR31*-binding sequence into psiCHECKTM downstream of the luciferase reporter gene. 293T cells transfected with psiCHECK-FGF3-wild-type had a 64.79% decrease (p<0.0001) in relative luciferase activity when cotransfected with the miR-31* mimic (Figure 4A). The changes in relative luciferase activity observed upon cotransfection of psiCHECK-FGF3-mutant with the miR-31* mimic or negative control was not significant (p>0.05). These findings suggest a direct interaction between miR-31* and the 3′ UTR of FGF3. On the other hand, changes in IL5Rα as measured by dual luciferase reporter assay were limited (Figure 4B). Furthermore, we performed quantitative reverse transcription- polymerase chain reaction (QRT-PCR) to examine the mRNA expression of FGF3 in the presence of miR-31* mimics/inhibitors. The decreased/increased mRNA expression of FGF3 was found when transfected with miR-31* mimics/inhibitors in Leuk-1, HIOEC and Cal-27 (Figure 5A). Western blot analyses showed that miR-31* mimics/inhibitors significantly attenuated/increased the FGF3 protein expressions in all three oral cell lines (Figure 5B). Together, these data suggest that FGF3 is a target gene of miR-31* and FGF3 expression is altered during OLK progression.

## Discussion

OLK is the most common premalignant lesion of the oral mucosa. To our knowledge, this is the first study that identifies alterations in miRNA expression between OLK and mtOLK. We found 25 upregulated miRNAs and nine downregulated miRNAs with greater than 2-fold changes in mtOLK. The miRNA profile identified was significantly different between OLK and mtOLK, providing evidence that miRNAs may serve as potential biomarkers in the process of OLK malignant transformation.

Among these altered miRNAs, miR-31* was chosen for further study for three reasons. First, miR-31* showed a significant fold change among the upregulated miRNAs. Also, this is the first report of altered miR-31* expression in cancer. Finally, miR-31* is derived from the same precursor with miR-31 and miR-31 was also overexpressed in the mtOLK samples studied. MiRNA*, the passenger strand of miRNA, is believed to be eliminated by cleavage or a bypass mechanism based on the ‘strand bias’ theory [10], [25]. However, recent studies have reported that many miRNA* species are actually relatively abundant and physically associated with effector complexes [11], [12], [13]. There is limited knowledge about miRNA*, especially its relationship to carcinogenesis. Therefore, our analyses of miR-31* in OLK and mtOLK elucidates the role of miRNA in OLK malignant transformation but also demonstrates the potential role of miRNA* in carcinogenesis.

Since a combination of different methods makes results more reliable [26], FISH was performed in the present study. These data consistently demonstrated miR-31* upregulation in mtOLK, but also allowed for visualization of the miR-31* signal. Therefore, FISH is a useful method to localize miRNA expression in frozen tissue sections [27].

In our study, all patients with a high FISH score, which included one OLK patient and three mtOLK patients, did not have recurrent or newly formed OLK during follow-up studies. In functional analysis, miR-31* greatly modulated the ability of apoptosis and migration in Leuk-1, HIOEC, and Cal-27 cells. However, in Cal-27 cells, miR-31* also modulated cell proliferation and cell cycle. Given these data, high miR-31* expression enhanced apoptosis and migration of potentially malignant cells as well as oral cancerous cells. The ability of miR-31* to modulate apoptosis and migration may be sufficient to prevent OLK recurrence or new leukoplakia growth.

MiR-31 has oncogenic functions in head and neck squamous cell carcinoma [9], [28]. Functional analyses of miR-31* may also broaden our understanding of miRNA* strands, especially those that are simultaneously expressed and paired miRNAs. Our results suggest that in addition to miR-31, miR-31* also has a biological function in cancer development.

Since establishing the multistep process of oral carcinogenesis, various oral keratinocyte cell lines have been used in the oral cellular model [22], [23]. Leuk-1 is spontaneously derived from an oral leukoplakia lesion that was adjacent to OSCC. It exhibits an immortalized but non-tumorigenic phenotype [22]. HIOEC is HPV16 E6/E7 immortalized oral keratinocyte cell line [23]. By induction with benzol[a]pyerene for 6 months, HIOEC cells become the cancerous cell line, HIOEC-BaP-96 (HB96) [24]. In our study, we used Leuk-1 and HIOEC cells, representing the precancerous stage in the cellular model. Indeed, miR-31* significantly modulated apoptosis and migration in Leuk-1 and HIOEC cells. In HIOEC cells, but in not Leuk-1 cells, miR-31* also affected cell proliferation. It suggests that miR-31* has similar but distinguishable effects in oral cell lines. The different effects in the various *in vitro* systems may be due to differences between organ systems and methods of immortalization (e.g., viral versus spontaneous) [22]. We used an oral cancer cell line, Cal-27, to represent the final stage in the cellular model of oral carcinogenesis. miR-31* modulated a variety of biological functions in Cal-27 cells, including cell proliferation, cell cycle, apoptosis, migration, and invasion. Taken together, miR-31* acted as a tumor suppressor associated gene in oral carcinogenesis. However, miR-31* may have different effects on biological function in oral cells with different malignant potential. Therefore, one should be cautious when addressing the roles of miR-31* in OLK malignant transformation. Given the notion that miR-31* is located in the cancer nest of mtOLK, further studies are needed to investigate whether the function of miR-31* is antagonistic to miR-31.

At present, the lack of knowledge regarding miRNA-targeted genes has hampered full understanding of cancer pathways that may be dysregulated by aberrant miRNA expression. To overcome this limitation, we used currently available computational approaches to predict gene targets. We found 655 candidate targets of miR-31* using the MicroCosm Targets program. These genes were involved in cytokine-cytokine receptor interactions, Chagas disease, the Jak-STAT signaling pathway, calcium signaling pathways, carbohydrate digestion and absorption, hematopoietic cell lineage determination, regulation of the actin cytoskeleton, and toxoplasmosis. We detected miR-31* expression in the inflammatory region and vascular area of mtOLK, which supports the hypothesis that miR-31* regulates genes related to cytokine-cytokine receptor interactions, Jak-STAT signaling, or hematopoietic cell lineage determination.

FGF3 was identified by its similarity with mouse fgf3/int-2, a proto-oncogene activated in virally induced mammary tumors in the mouse [29]. The protein encoded by this gene is a member of fibroblast growth factor family and plays a role in various processes such as cell proliferation, angiogenesis, and embryogenesis [30]. In normal human breast tissue, expression of this gene is undetectable and it is expressed at low levels in the healthy brain and testis [31]. Ectopic expression of FGF3 in prostate epithelial cells elicits rapid hyperplasia [32]. In the FGF3 stably transfected mouse epithelial cell line, FSK4, expression of FGF3 causes proliferative changes, including reduced contact inhibition, increased β-catenin expression, and decreased p53 transcriptional activity [33]. Hajitou *et al*. found that FGF3-transfected EF43 mouse cells were strongly invasive in matrigel-coated chambers and caused tumor metastases in nude mice [34]. Hu *et al*. used a hepatocellular carcinoma tissue microarray and found that FGF3 overexpression is significantly associated with hepatocellular carcinoma metastasis and recurrence, suggesting that FGF3 upregulation may play an important role in these processes [35]. Studies have also shown amplification of FGF3 in nasopharyngeal carcinoma [36], ovarian cancer [37], endometrial carcinomas [38], lung cancer [39], and oral cancer [40], [41], [42], [43], which may be important for neoplastic transformation and tumor progression. Here, we first provide evidence that the 3′ UTR of FGF3 physically combines with miR-31*. We also showed that miR-31* could negatively regulate FGF3 expression level in oral cells. It suggests that miR-31* may play a role through regulating FGF3 during OLK progression. We noticed that FGF3 expression was decreased but not completely ablated when over-expressing miR-31* in Cal-27. This data indicates that regulating of FGF3 expression may involve multiple factors and miR-31* is one of the effecter to down regulate FGF3.

Taken together, upregulation of miR-31* is negatively correlated with recurrent/newly formed OLK. MiR-31* may exert different effects on biological function in oral cells with different malignant potential. FGF3 is the target of miR-31* and miR-31* may play an important role during OLK progression through regulating FGF3. Our data further suggest that miRNA* strands may also have prominent roles in oral carcinogenesis.

## Materials and Methods

### Patients and Samples

Twenty-seven tissue samples (20 OLK and seven mtOLK) were collected from patients of the Department of Oral Mucosal Diseases and the Department of Oral and Maxillofacial Surgery, Ninth People’s Hospital, Shanghai Jiao Tong University School of Medicine. The diagnosis of OLK, mtOLK, and epithelial dysplasia were made according to World Health Organization criteria [27]. Tumor-node-metastasis classification [44] was also applied to mtOLK patients. Baseline characteristics of the patients are shown in Table 2. To identify changes in miRNA expression between OLK and early-stage mtOLK, all mtOLK patients enrolled in the study were classified as T_1_N_0_M_0._ The mean follow-up period was 32 months (from 20 to 47 months). All mtOLKs were diagnosed as squamous cell carcinoma upon collection, with a history of OLK at the same site. This study was approved by the Human Research Ethics Committee of Shanghai Ninth People’s Hospital, Shanghai Jiao Tong University School of Medicine (#200703). Informed written consent was obtained from all patients before sampling.

**Table 2:** Baseline characteristics of the subjects studied.

	OLK (20)	mtOLK (7)	*P*
Gender			0.678
Male	11	3	
Female	9	4	
Age (years) mean ± SD	59.00±9.33	55.43±16.33	0.564
Smoking			1.000
Never/Former	11	4	
Current	9	3	
Alcohol consumption			1.000
Never/Former	8	3	
Current user	12	4	
Epithelial dysplasia			
None/Mild	11	–	–
Moderate	5	–	–
Severe	4	–	–
TNM classification			
T_1_N_0_M_0_	–	7	–

OLK, oral leukoplakia; mtOLK, malignant transformed oral leukoplakia; SD, standard deviation; TNM, tumor-node-metastasis.

### RNA Isolation and miRNA Microarray

Total RNA was isolated from all samples using TRIZOL reagent (Invitrogen, USA) according to the manufacturer’s protocol. We employed miRCURY™ Locked Nucleic Acid Array version 11.0 (Exiqon, Denmark), which contains capture probes for all human miRNAs (847 total), to identify the miRNA expression profile in OLK tissues, as previously described [45]. Scanning was performed using the Axon GenePix 4000B microarray scanner (Axon Instruments, USA). GenePix Pro 6 was used to read the raw intensity of the image. The ratio of red signals to green signals was calculated after background subtraction and normalization using the global Lowess regression algorithm. Significant miRNAs were selected and unsupervised hierarchical clustering was also performed on the miRNA expression profile. All data is Minimum Information About a Microarray Experiment-compliant and the raw data has been deposited in a Minimum Information About a Microarray Experiment-compliant database (GEO), as detailed on the Microarray Gene Expression Data Society website. The accession number is GSE33299 and the website address is www.ncbi.nlm.nih.gov/geo.

### FISH Detection

FISH was performed as previously described, with some modifications [46]. In brief, 10– µm frozen tissue sections were fixed and pre-hybridized at 51°C. Locked nucleic acid-modified and fluorescein isothiocyanate-labeled oligonucleotide probes (3 pmol; Exiqon) were hybridized to the sections at 51°C. The sections were incubated with a fluorescein isothiocyanate/horseradish peroxidase primary antibody (DAKO, Denmark). Signals were detected using the tyramide signal amplification system (Perkin-Elmer, USA) and analyzed with Image-Pro Plus software. The FISH score was based on both the intensity of staining and percentage of positive cells. The scale for staining intensity was 0 (negative), 1 (weak), or 2 (strong). The percentage of positive cells was defined as 0 (0%), 1 (interspersed or 0–10%), 2 (focal or 10–50%), or 3 (diffuse or >50%). A “FISH score” was generated as the product of intensity times area, similar to what has been previously described [47].

### Cell Culture and Transfection

The human oral leukoplakia cell line Leuk-1 was kindly provided by Dr. Li Mao (MD Anderson Cancer Center, USA). It is spontaneously derived from an oral leukoplakia lesion that was adjacent to OSCC. It exhibits an immortalized but non-tumorigenic phenotype [7]. The human immortalized oral epithelial cell line, HIOEC, was established by the Shanghai Key Laboratory of Stomatology, which was obtained from normal oral mucosa immortalized with HPV16 E6/E7 gene transfection. Both of these cell lines were cultured in keratinocyte media-serum-free media (Gibco, USA). The oral cancer cell line Cal-27 (American Type Culture Collection, USA) was grown in Dulbecco’s modified Eagle medium (Gibco). The miR-31* mimic (C-301029-01-0005), inhibitor (IH-301029-02-0005), and negative control (IN-001005-01-05) were all purchased from Thermo Scientific Dharmacon (USA). Cells were transfected 24 h after plating using Lipofectamine 2000 reagent (Invitrogen). Transfection complexes were prepared according to the manufacturer’s instructions.

### MTT Assays

Cells were seeded in 96-well plates at a density of 5000 cells/well. At various time points post-transfection (24 h, 48 h, and 72 h), 20 µl of 3-(4, 5-dimethylthiazol-2-yl)-2, 5-diphenyltetrazolium bromide (MTT) was added to the test well and incubated for 4 h. The supernatant was then discarded and 200 µl of dimethyl sulfoxide was added. Absorbance was measured at a wavelength of 490 nm using a microplate reader (Tecan Sunrise, Austria). Each experiment was repeated in triplicate.

### Cell Cycle Analyses

Parental and transfected cells in the log phase of growth were stained with propidium iodide [48] and examined using a FACScan flow cytometer (BD Biosciences, USA). DNA histograms were analyzed with ModFit LT for Mac V 3.1 (Verity Software House, USA).

### Annexin V Assays

Apoptosis was measured using an Annexin V/7-AAD flow cytometric assay (BD Biosciences) according to the manufacturer’s instructions and then analyzed using a FACScan flow cytometer. Data analyses were performed using Cell-Quest software (BD Biosciences). All experiments were run in triplicate.

### Cell Migration and Invasion Assays

After transfection, cells were incubated for 12 h in serum-free media. Transwell inserts with 8– µm pores were purchased from Millipore Corporation (USA). Cells (20×10^5^) were resuspended in the upper chamber in 200 µl serum-free media and incubated for 16 h for the migration assay. For the invasion assay, 8.0×10^5^ cells in 200 µl serum-free media were added to the matrigel-coated upper chamber and incubated for 48 h. Cells on the lower surface of the insert were Giemsa (Cal-27 and Leuk-1) or Coomassie brilliant blue (HIOEC) stained and images from five representative fields of each membrane were taken using a light microscope (100× magnification). The number of migratory or invasive cells was counted. Each experiment was repeated three times.

### Bioinformatics

The following online software programs were used to seek candidate targets of miR-31*: PicTar (http://pictar.bio.nyu.edu), TargetScan 4.1 (http://www.targetscan.org), MicroCosm Targets Version 5 (http://www.ebi.ac.uk/enright-srv/microcosm/cgi-bin/targets/v5/search.pl), and miRanda (http://cbio.mskcc.org/cgi-bin/mirnaviewer/mirnaviewer.pl). The MicroCosm Targets software program was the only one to show candidate targets of miRNA-31*. GeneCodis 2.0 [49], [50] was then used to search for biological features and pathway enrichment analyses of the interacting genes involved in the network.

### Plasmid Construction

To evaluate the binding of miR-31* to FGF3, its 3′UTR (NM-005247) was amplified by PCR using the primers 5′-GCCTCGAGACCTGGAGCCCTCTCACGTT-3′ and 5′-CAGCGGCCGCGCTGTGCTGGAAGCAAGCAC-3′. The product was cloned into XhoI and NotI sites of the psiCHECKTM-2 vector (Promega, USA) for subsequent luciferase assays. Mutations on the complementary site were introduced by PCR mutagenesis using the QuikChange site-directed mutagenesis kit protocol (Stratagene, USA) and were confirmed by DNA sequencing. The 3′ UTR (NM-000564) of IL5Rα was also amplified by PCR using the primers 5′-GCCTCGAGGCCTGGAGTTGAGACCCTGG-3′ and 5′-CAGCGGCCGCGGGGGTGAGGAATTTGTGGCT-3′. The product was then cloned into psiCHECKTM-2 as described above.

### Dual Luciferase Reporter Assays

293T cells were seeded at a density of 2.5×10^4^ cells/well in a 12-well plate and grown for 48 h. A total of 400 ng of the plasmids (psiCHECK-FGF3-wild type/psiCHECK -IL5Rα -wild type and psiCHECK-FGF3-mutant/psiCHECK -IL5Rα -mutant) were cotransfected with 50 pmol miR-31* using Lipofectamine 2000 (Invitrogen). After 24 h, cell extracts were obtained and firefly and renilla luciferase activities were measured with the dual-luciferase reporter system (Promega) according to the manufacturer’s instructions.

### QRT-PCR

A two-step QRT-PCR was performed using SuperScript® Reverse Transcription Kits (Invitrogen, USA) and SYBR® Green Master Mixes (Invitrogen, USA) according to the manufacturer’s instructions. In brief, the total RNA of every sample was converted into cDNA using random primer. Then quantitative PCR was performed in a Step-One Plus Real-Time PCR System (Applied Biosystems, USA). The reaction conditions were applied according to the manufacturer’s manually, which were 95°C for 2 min, followed by 40 cycles of 94°C for 10 sec and 58°C for 10 sec and 72°C for 40 sec. All samples were completed in triplicate. The GAPDH was used as internal control and the relative expression level of FGF3 was determined using comparative CT method (2^-ΔΔCT^). The following primers were used: for FGF3, 5′- GTACCACCTCCAGCTGCACC -3′ (forward) and 5′- GCGTACTAGACACCGTCCGG -3′ (reverse); and for GAPDH, 5′- AATTCCATGGCACCGTCAAG -3′ (forward) and 5′- TGGTTCACACCCATGACGAA -3′ (reverse).

### Western Blotting

Cell extracts were prepared in lysis buffer and centrifuged at 12,000×*g* at 4°C. The total protein concentration was measured using a bicinchoninic acid assay. Cellular extracts containing 50 µg total protein were analyzed using 10% sodium dodecyl sulfate polyacrylamide gel electrophoresis and transferred electrophoretically to polyvinylidene difluoride membranes (Invitrogen, USA). Blots were probed at 4°C overnight with primary antibodies in 5% milk/TBST. The antibodies used for western blotting were FGF3 and GAPDH (Abcam Ltd., UK).

### Statistical Analyses

Student’s t-tests for two groups or one-way analysis of variance were performed to evaluate the statistical significance of clustered miRNAs in each group as well as in the functional assays. The χ^2^ test was used to analyze the difference in the rate of FISH between OLK and mtOLK. Fisher’s exact tests and logistic regression models were used to analyze the relationship between clinical parameters and miRNA. Data analyses were performed using SPSS for Windows version 16.0 (SPSS Inc., USA). Tests were two-sided and p<0.05 was considered statistically significant.

## Supporting Information

Figure S1 Effects on biological functions of miR-31* in Leuk-1. (**A**) the MTT assay in Leuk-1; (**B**) the Annexin V assay in Leuk-1; (**C**) flow cytometry analysis for cell cycle in Leuk-1; (**D**) representative field of view of Leuk-1 invasion inserts at a 100× magnification, (Da) miR-31* mimic, (Db) miR-31* inhibitor, (Dc) negative control, (Dd) Quantification of relative numbers of invading cells representing average counts from 6 fields-of-view per insert per sample ±SD, **p<0.05, ***p<0.001; (**E**) representative field of view of Leuk-1 migration inserts at a 100× magnification, (Ea) miR-31* mimic, (Eb) miR-31* inhibitor, (Ec) negative control, (Ed) quantification of relative numbers of invading cells representing average counts from 6 fields-of-view per insert per sample ±SD, **p<0.05, ***p<0.001.
(TIF)

Figure S2 Effects on biological functions of miR-31* in HIOEC. (**A**) the MTT assay in HIOEC; (**B**) the Annexin V assay in HIOEC; (**C**) flow cytometry analysis for cell cycle in HIOEC; (**D**) representative field of view of HIOEC invasion inserts at a 100× magnification, (Da) miR-31* mimic, (Db) miR-31* inhibitor, (Dc) negative control, (Dd) Quantification of relative numbers of invading cells representing average counts from 6 fields-of-view per insert per sample ±SD, **p<0.05, ***p<0.001; (**E**) representative field of view of HIOEC migration inserts at a 100× magnification, (Ea) miR-31* mimic, (Eb) miR-31* inhibitor, (Ec) negative control, (Ed) quantification of relative numbers of invading cells representing average counts from 6 fields-of-view per insert per sample ±SD, **p<0.05, ***p<0.001.
(TIF)

Figure S3 Effects on biological functions of miR-31* in Cal-27. (**A**) the MTT assay in Cal-27; (**B**) the Annexin V assay in Cal-27; (**C**) flow cytometry analysis for cell cycle in Cal-27; (**D**) representative field of view of Cal-27 invasion inserts at a 100× magnification, (Da) miR-31* mimic, (Db) miR-31* inhibitor, (Dc) negative control, (Dd) Quantification of relative numbers of invading cells representing average counts from 6 fields-of-view per insert per sample ±SD, **p<0.05, ***p<0.001; (**E**) representative field of view of Cal-27 migration inserts at a 100× magnification, (Ea) miR-31* mimic, (Eb) miR-31* inhibitor, (Ec) negative control, (Ed) quantification of relative numbers of invading cells representing average counts from 6 fields-of-view per insert per sample ±SD, **p<0.05, ***p<0.001.
(TIF)

Table S1 The related pathways of the targeted genes of miR-31*.
(DOC)
