# Supplementary figures and images for: Upregulation of miR-31* Is Negatively Associated with Recurrent/Newly Formed Oral Leukoplakia
Source: PLoS One. 2012 Jun 18;7(6):e38648. doi: 10.1371/journal.pone.0038648 (PMC3377716; doi:10.1371/journal.pone.0038648)

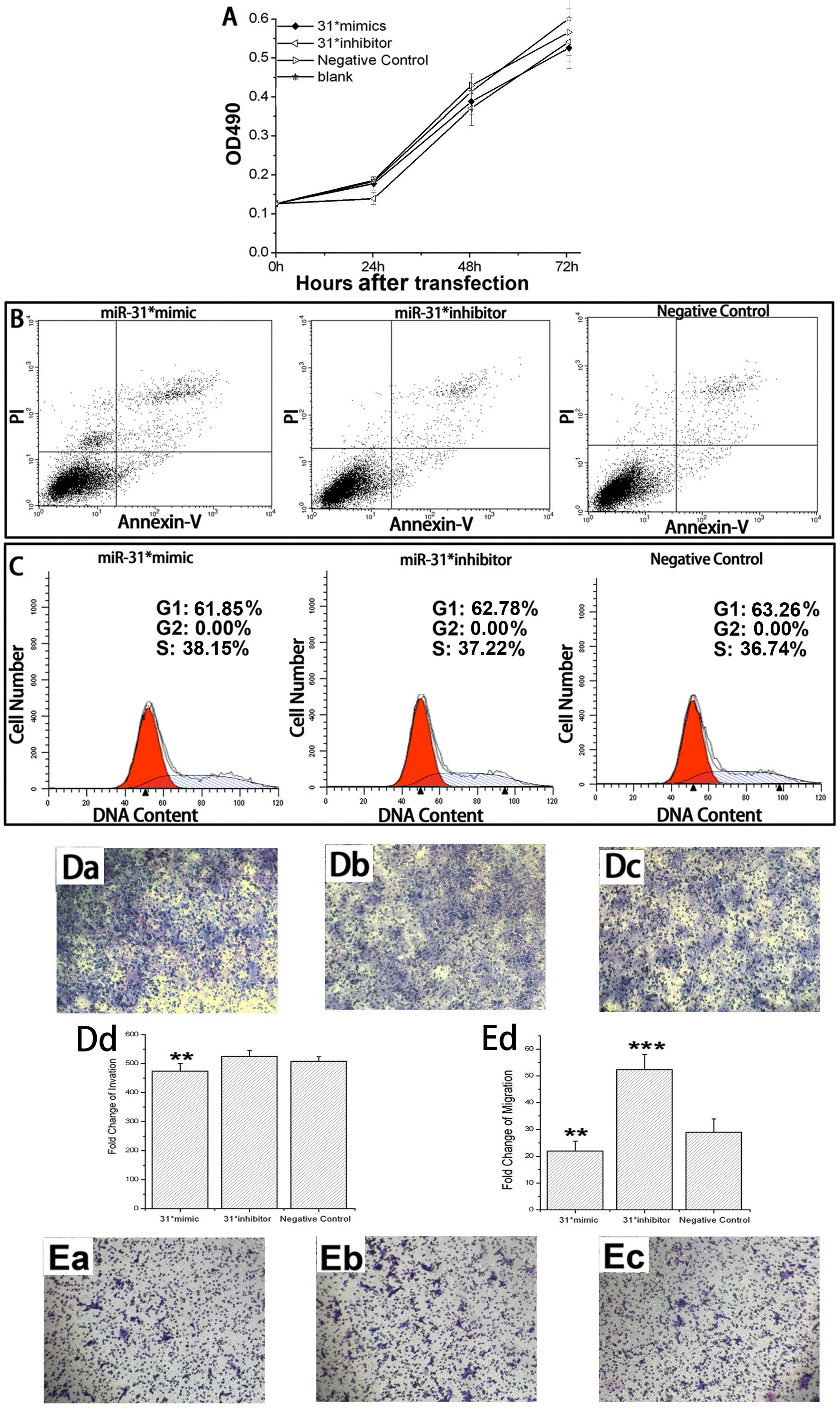

Supplement: Figure S1 — Effects on biological functions of miR-31* in Leuk-1. (A) the MTT assay in Leuk-1; (B) the Annexin V assay in Leuk-1; (C) flow cytometry analysis for cell cycle in Leuk-1; (D) representative field of view of Leuk-1 invasion inserts at a 100× magnification, (Da) miR-31* mimic, (Db) miR-31* inhibitor, (Dc) negative control, (Dd) Quantification of relative numbers of invading cells representing average counts from 6 fields-of-view per insert per sample ±SD, **p<0.05, ***p<0.001; (E) representative field of view of Leuk-1 migration inserts at a 100× magnification, (Ea) miR-31* mimic, (Eb) miR-31* inhibitor, (Ec) negative control, (Ed) quantification of relative numbers of invading cells representing average counts from 6 fields-of-view per insert per sample ±SD, **p<0.05, ***p<0.001. (TIF) [file pone.0038648.s001.tif]

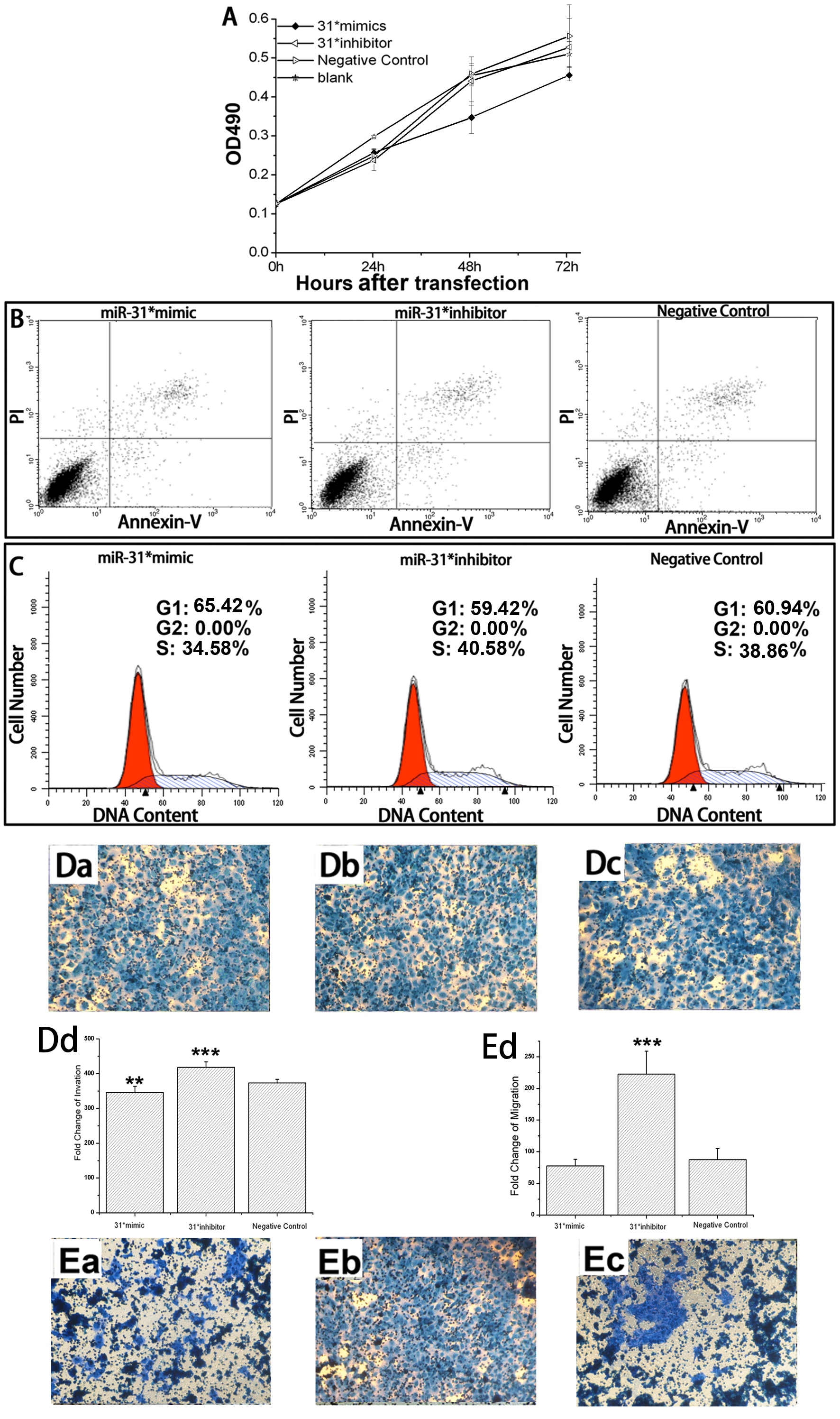

Supplement: Figure S2 — Effects on biological functions of miR-31* in HIOEC. (A) the MTT assay in HIOEC; (B) the Annexin V assay in HIOEC; (C) flow cytometry analysis for cell cycle in HIOEC; (D) representative field of view of HIOEC invasion inserts at a 100× magnification, (Da) miR-31* mimic, (Db) miR-31* inhibitor, (Dc) negative control, (Dd) Quantification of relative numbers of invading cells representing average counts from 6 fields-of-view per insert per sample ±SD, **p<0.05, ***p<0.001; (E) representative field of view of HIOEC migration inserts at a 100× magnification, (Ea) miR-31* mimic, (Eb) miR-31* inhibitor, (Ec) negative control, (Ed) quantification of relative numbers of invading cells representing average counts from 6 fields-of-view per insert per sample ±SD, **p<0.05, ***p<0.001. (TIF) [file pone.0038648.s002.tif]

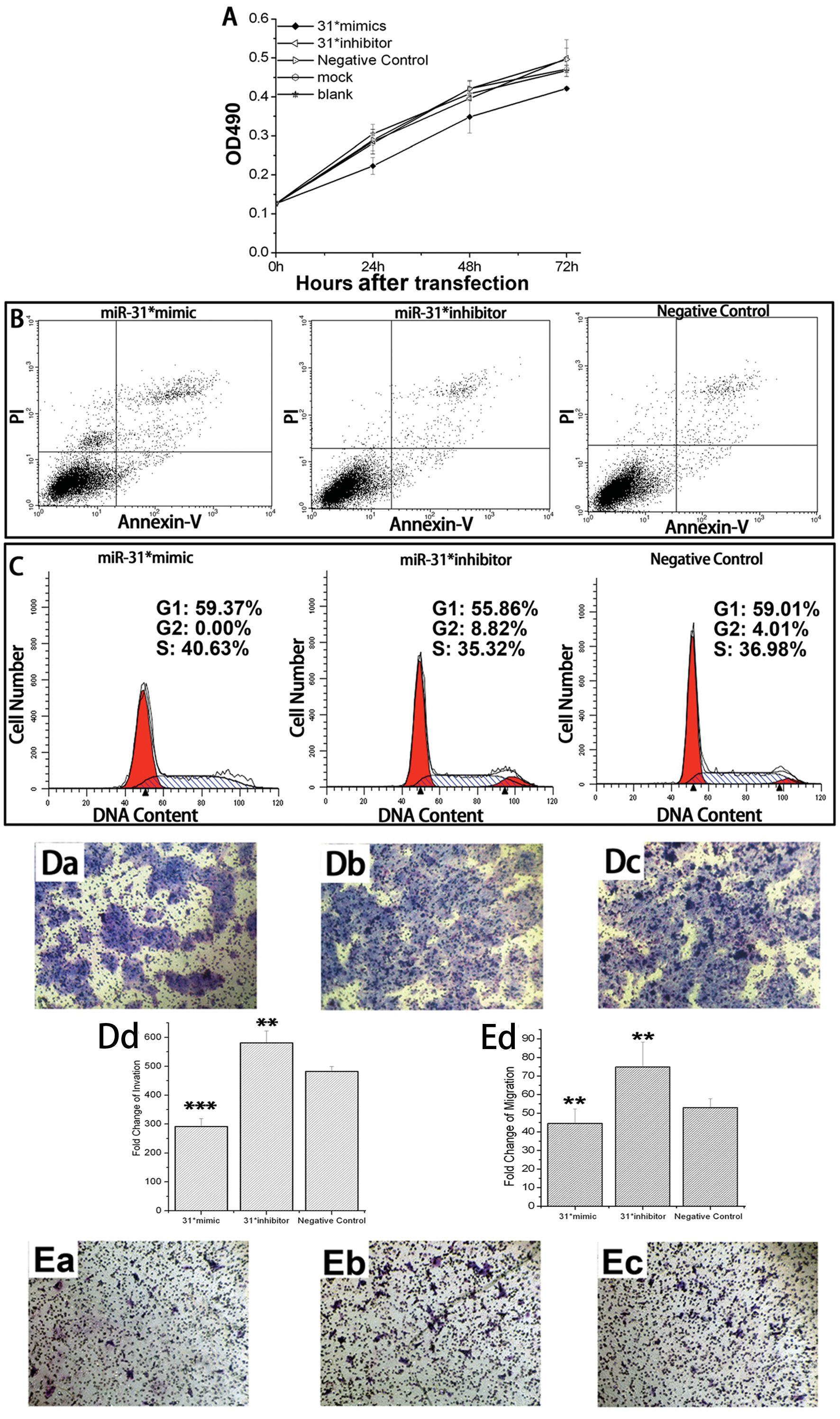

Supplement: Figure S3 — Effects on biological functions of miR-31* in Cal-27. (A) the MTT assay in Cal-27; (B) the Annexin V assay in Cal-27; (C) flow cytometry analysis for cell cycle in Cal-27; (D) representative field of view of Cal-27 invasion inserts at a 100× magnification, (Da) miR-31* mimic, (Db) miR-31* inhibitor, (Dc) negative control, (Dd) Quantification of relative numbers of invading cells representing average counts from 6 fields-of-view per insert per sample ±SD, **p<0.05, ***p<0.001; (E) representative field of view of Cal-27 migration inserts at a 100× magnification, (Ea) miR-31* mimic, (Eb) miR-31* inhibitor, (Ec) negative control, (Ed) quantification of relative numbers of invading cells representing average counts from 6 fields-of-view per insert per sample ±SD, **p<0.05, ***p<0.001. (TIF) [file pone.0038648.s003.tif]
