# Supplementary material for: Upregulation of miR-31* Is Negatively Associated with Recurrent/Newly Formed Oral Leukoplakia
Source: PLoS One. 2012 Jun 18;7(6):e38648. doi: 10.1371/journal.pone.0038648 (PMC3377716; doi:10.1371/journal.pone.0038648)
Supplement: Table S1 — The related pathways of the targeted genes of miR-31*. (DOC) [file pone.0038648.s004.doc]

**Supporting Information Table**

**Table S1. The related pathways of the targeted genes of miR-31*.**

| Number | Related to pathways | Genes |
| --- | --- | --- |
| 17 | Cytokine-cytokine receptor interaction | IL5Rα,IL15,IFNGR1,FLT3LG,TNFSF18,TNFSF11,MPR1B,ACVR1,LTB,IFNB1,OSMR,CRLF2,  IL11Rα,CSF2,CD70,IL7,CD40 |
| 9 | Chagas disease (American trypanosomiasis) | IFNGR1,TLR2,IKBKG,MAPK13,GNAL,PPP2CA,BDKRB2,IFNB1,PIK3CG |
| 11 | Jak-STAT signaling pathway | IL5Rα,IL15,IFNGR1,IFNB1,OSMR,CRLF2,STAT3,IL11Rα,CSF2,IL7,PIK3CG |
| 11 | Calcium signaling pathway | PTAFR,VDAC1,TRPC1,ITPR1,CACNA1S,PTGER3,GNAL,PLCZ1,BDKRB2,PHKA2,GRM5 |
| 5 | Carbohydrate digestion and absorption | HK1,ATP1A1,MGAM,PIK3CG,SLC2A5 |
| 7 | Hematopoietic cell lineage | IL5Rα,ANPEP,FLT3LG,DNTT,IL11RA,CSF2,IL7 |
| 11 | Regulation of actin cytoskeleton | TIAM2,SLC9A1,ARHGEF1,ITGAD,RHOA,ITGB2,FGF3,ROCK1,BDKRB2,ACTB,PIK3CG |
| 8 | Toxoplasmosis | IFNGR1,TLR2,IKBKG,MAPK13,LAMB4,STAT3,PIK3CG,CD40 |
